# Cross-Linked Dependency of Boronic Acid-Conjugated Chitosan Nanoparticles by Diols for Sustained Insulin Release

**DOI:** 10.3390/pharmaceutics8040030

**Published:** 2016-10-08

**Authors:** Nabil A. Siddiqui, Nashiru Billa, Clive J. Roberts, Yaa Asantewaa Osei

**Affiliations:** 1 School of Pharmacy, University of Nottingham Malaysia Campus, Jalan Broga, Semenyih 43500, Selangor Darul Ehsan, Malaysia; khyx5naq@nottingham.edu.my; 2 School of Pharmacy, University of Nottingham, University Park, Nottingham, NG7 2RD, UK; Clive.Roberts@nottingham.ac.uk; 3 Faculty of Pharmacy and Pharmaceutical Sciences, Kwame Nkrumah University of Science and Technology, Kumasi, Ghana; yaosei.pharm@knust.edu.gh

**Keywords:** stimuli-responsive, glucose, fructose, drug delivery, nanoparticles

## Abstract

Boronic acids have been widely investigated for their potential use as glucose sensors in glucose responsive polymeric insulin delivery systems. Interactions between cyclic diols and boronic acids, anchored to polymeric delivery systems, may result in swelling of the delivery system, releasing the drug. In this study, 4-formylphenylboronic acid conjugated chitosan was formulated into insulin containing nanoparticles via polyelectrolyte complexation. The nanoparticles had an average diameter of 140 ± 12.8 nm, polydispersity index of 0.17 ± 0.1, zeta potential of +19.1 ± 0.69 mV, encapsulation efficiency of 81% ± 1.2%, and an insulin loading capacity of 46% ± 1.8% *w*/*w*. Changes in size of the nanoparticles and release of insulin were type of sugar- and concentration-dependent. High concentration of diols resulted in a sustained release of insulin due to crosslink formation with boronic acid moieties within the nanoparticles. The formulation has potential to be developed into a self-regulated insulin delivery system for the treatment of diabetes.

## 1. Introduction

Biocompatible and biodegradable polymers have attracted much attention by formulation scientists in recent years. Advancements in polymer science and nanotechnology have made it possible for these polymers to be suitably modified with biological and chemical entities for stimuli responsive and targeted drug delivery [1,2,3]. Chitosan is one such polymer which is synthesized by treating chitin (a natural cellulose derivative commonly present in crustaceans such as crabs and shrimps) in alkaline media. It is mucoadhesive, biocompatible [4] and promotes the passage of biomolecules across biological epithelia. As a result, it has been studied for potential use in various pharmaceutical dosage forms including beads, microparticles and nanoparticles [5,6,7]. Recently, various researchers have successfully introduced stimuli-responsive moieties such as concavalin A [8], glucose oxidase [9] and boronic acids [10] to chitosan with the aim to regulate the release of insulin from their delivery systems. This pursuit fits well with the quest for appropriate management of Type 1 diabetes, which necessitates repeated and life-long subcutaneous injections of insulin. 

Current insulin replacement therapy for the management of diabetes involves injections of fast-acting insulin during mealtimes, followed by longer-acting insulin injections which maintain a baseline insulin level throughout the day [11,12]. Subcutaneous injections of insulin are painful and cumbersome leading to poor patient compliance. To overcome these challenges several new technologies have been developed which include insulin pumps. However, they too have drawbacks such as frequent maintenance, risk of infection and inflammation. Future insulin therapies need to be glucose-regulated, less painful, relatively inexpensive to manufacture and easily available for administration in clinical settings [13]. 

In the last two decades, advances in nanotechnology have vastly improved both diagnosis and treatments in the field of cancer and cardiovascular biology [14,15,16,17]. Various nanoparticulate formulations such as liposomes, polymer nanoparticles, nanostructures, metallic nanoparticles and stimuli-responsive nanoparticles have proved to be not only biocompatible but also possess ideal physicochemical properties for potential biomedical applications [18,19,20,21,22]. Using nanotechnology, drugs can be delivered to the site of action only, thereby reducing systemic side-effects and cost of treatment. In the recent past, scientists have started exploring the potential of nanotechnology for diagnosis, monitoring and treatment of diabetes. Progress in the field of nanotechnology and polymer science can now enable scientists to engineer nanoparticles that can release the loaded drug by sensing changes in their surroundings [23]. Currently, the three most extensively studied glucose sensors are glucose oxidase, glucose-binding proteins (GBPs) such as lectins, and glucose-binding small molecules such as boronic acids (BAs). In the present context, nanoparticles are selected due to the high surface area to volume ratio, which offers rapid reaction time capability.

BAs bind reversibly to *cis*-1,2- or 1,3-diols via covalent interactions to form five- or six-membered cyclic esters. The interaction has been proven to be so strong that mM or even sub-mM levels of saccharides in biological systems can bind to boronic acids. While BAs offer the advantage of being oxygen-independent (unlike glucose oxidase), elicit no immunological responses (unlike GBPs) and have fast response rates, they lack in selectivity for diols. Research is underway to improve the sensitivity of boronic acids to glucose [23]. Over the last ten years, scientists have proposed several methods to develop glucose-specific boronic acid-based sensors. Currently, four main strategies are being widely investigated which include synthetic diboronic acids, boronic acid-conjugated polymers, self-assembly of simple boronic acids and boronic acid-conjugated nanomaterials [24]. Asantewaa et al., studied various physicochemical properties of chitosan conjugated to 4-formylphenylboronic acid and also investigated how this modification of the polymer influenced its propensity to glucose sensing [25].

Presently, our aim is to formulate one of those boronic acid-functionalised chitosan conjugates into a glucose responsive insulin-containing nanoparticulate delivery system and ascertain its physicochemical properties in response to changes in sugar (glucose and fructose) concentrations. Various approaches have been utilised to encapsulate peptide and protein molecules including ionotropic gelation [26], polyelectrolyte complexation (PEC) [27] and layer-by-layer adsorption [28]. In our current investigation, a nanoparticulate insulin delivery system has been prepared via PEC with special attention paid to the encapsulation efficiency and insulin release of the delivery system in response to two main physiological diols (glucose and fructose). It is envisaged that successful development of the proposed formulation would enable the delivery of insulin on a less frequent basis via a variety of possible routes such as transdermal patches, depot injections, inhalable devices or even oral routes. This will eliminate the need for repeated invasive administration, while at the same time significantly improving overall glycaemic control of insulin therapy.

## 2. Experimental Section

### 2.1. Materials

Low molecular weight chitosan was purchased from Sigma Aldrich (St. Louis, MO, USA); sodium tripolyphosphate (TPP), 4-formylphenylboronic acid and sodium borohydride from Thermo Fischer Scientific (Bridgewater, NJ, USA); acetic acid, methanol, acetonitrile, human recombinant zinc insulin from *P. pastoris* (28.5 IU/mg) , fructose and glucose were purchased from Merck (Whitehouse, NJ, USA). All other chemicals were of reagent grade.

### 2.2. Methods

#### 2.2.1. Synthesis of Chitosan-Phenylboronic Acid Conjugates 

Chitosan (400 mg) dissolved in 1% acetic acid solution was reacted with 360 mg (2.4 mmol) of 4-formylphenylboronic acid (PBA) (dissolved in methanol) in the presence of sodium borohydride as the reducing agent. The reaction was maintained at room temperature and made to run for 24 h. The resulting PBA-bonded chitosan conjugate (labeled as F1 through F5, Table 1) was separated from the reaction mixture by centrifugation at 6000 rpm for 10 min and washed with methanol, ethanol and water. The conjugate was then freeze-dried and stored at 2–8 °C until used.

#### 2.2.2. Preparation Insulin-Containing Nanoparticles from Conjugates

Chitosan conjugate (F3) was dissolved in 1% acetic acid to a concentration of 2 mg/mL and the pH was adjusted to 5.5 with 1 M NaOH. A 1 mg/mL solution of insulin was prepared in a mixture of 0.01 M HCl and 0.1 M Tris base at a ratio of 87:13 and the pH was adjusted to 8.5 with 1 M NaOH. 1 mL of the functionalized chitosan solution was measured into a vial and 2 mL of the insulin solution was added drop-wise whilst stirring at 600 rpm on a magnetic stirrer for 40 min. The temperature of the mixture was maintained at 25 °C and the mixture was visually inspected, where an opalescent dispersion was indicative of nanoparticle formation. The nanoparticulate formulation was designated F3PN.

#### 2.2.3. Physical Characterization of Nanoparticles

The size (z-average), polydispersity index (pdi) and charge (zeta potential) of the nanoparticles were assessed using a Zetasizer Nano ZS (Malvern, UK) equipped with a 4 mW He-Ne laser (633 nm). Each analysis was carried out at 25 °C, performed in triplicate and the data expressed as mean ± standard deviation. The morphology and surface topography of nanoparticles was performed on a field emission scanning electron microscope (FESEM, Quanta 400F, FEI Company, Fremont, CA, USA) under low vacuum and at a viewing voltage of 20.0 kV. After a 1:10 dilution with deionized water, a drop of freshly prepared nanoparticulate solution was placed onto an SEM imaging stub and left to air-dry at room temperature for 24 h before viewing.

#### 2.2.4. HPLC Analysis

The amount of insulin in the samples was detected using an Agilent HPLC system (Agilent Technologies, CA, USA) equipped with a UV detector. The mobile phase consisted of 0.1% aqueous triflouroacetic acid (TFA) (A) and 0.1% TFA in acetonitrile (B). A gradient elution was used with the mobile phase starting with 75% of A and decreasing to 40% over 8 min at a flow rate of 1 mL/min. The injection volume was 20 µL and the detection wavelength 214 nm. The column was Agilent Zorbax 300SB-4.6mm × 250 mm C_18_, with particle size of 5 μm and pore size of 300 Å. The calibration curve was constructed from pure insulin dissolved in 0.01 M HCl at a range of 0.625–100 μg/mL.

#### 2.2.5. Evaluation of Encapsulation Efficiency and Loading Capacity of Nanoparticles

The encapsulation efficiency (EE%) and loading capacity (LC) of insulin within the nanoparticles (F3PN) were determined upon separation of the nanoparticles from the aqueous medium containing free insulin by centrifugation at 14,000 rpm for 45 min using a Beckman Coulter Microfuge 16 centrifuge (Beckman Coulter, Brea, CA, USA). The amount of free insulin in the supernatant was measured using the above HPLC procedure by comparing peak area obtained with that from the calibration curve. All samples were run in triplicate. The EE% and LC for insulin were calculated as:

(1) $$\mathrm{EE}\%=\;\frac{\mathrm{total}\;\mathrm{insulin}\;\mathrm{in}\;\mathrm{formulation}-\mathrm{free}\;\mathrm{insulin}\;\mathrm{in}\;\mathrm{supernatant}}{\mathrm{total}\;\mathrm{insulin}\;\mathrm{in}\;\mathrm{formulation}}\;\times 100$$

(2) $$\mathrm{LC}\%=\;\frac{\mathrm{total}\;\mathrm{insulin}\;\mathrm{in}\;\mathrm{formulation}-\mathrm{free}\;\mathrm{insulin}\;\mathrm{in}\;\mathrm{supernatant}}{\mathrm{weight}\;\mathrm{of}\;\mathrm{nanoparticles}}\;\times 100$$

#### 2.2.6. In Vitro Insulin Release Studies as a Function of Diol Concentration

The amount of insulin released from the nanoparticles was studied as a function of glucose and fructose concentrations (1, 3 and 5 mg/mL). Several 100 µL replicas of F3PN were placed in Eppendorf tubes containing 1 mL of the various release media and incubated at 37 °C with horizontal shaking at 100 rpm on a WiseCube WIS-20, Precise Shaking Incubator (Witeg, Wertheim, Germany). At predetermined time points, one of the seeded tubes was withdrawn and centrifuged at 14,000 rpm for 15 min using a Beckman Coulter Microfuge (Beckman Coulter, Brea, CA, USA) 16 centrifuge which was followed by injection of 20 μL of the supernatant into the HPLC system. The amount of insulin released in the respective media was computed by comparing peak area obtained with those from the calibration curve.

#### 2.2.7. Size Changes of Nanoparticles as a Function of Glucose Concentration

The size changes of the nanoparticles as a function of glucose concentration were studied using a nanoparticle tracking analysis (NTA) equipment (Nanosight LM 10 Nanosight, Amesbury, UK). The equipment was calibrated using polystyrene beads solution over 1, 2.5, 5 and 10 mg/mL of glucose. 1 mL of each glucose solution was measured into a small clean vial followed by the addition of 1 μL of polystyrene bead solution. A 0.3 mL aliquot of the mixture was then injected into the viewing chamber (equipped with a 405 nm laser wavelength) with a sterile syringe until the liquid reached the tip of the nozzle. All measurements were performed at room temperature. Videos lasting for 1 min 30 s were captured at 30 frames per second and analysed. The same procedure was applied to determine the size changes of F3PN after exposure to various glucose solutions. Size changes of F3PN (100 µL of freshly prepared samples) in fructose and glucose as a function of time at concentrations of 1, 3 and 5 mg/mL (1 mL each) were studied in a capped Zetasizer cuvette. Z-average in each medium was recorded over 60 min.

#### 2.2.8. Statistical Analysis

A simple two-tailed *t*-test was performed with 95% confidence interval to check for significant differences between experimental results where necessary.

## 3. Results and Discussion

### 3.1. Formulation of Insulin-Loaded Functionalised Chitosan Nanoparticles

**Figure 1:**
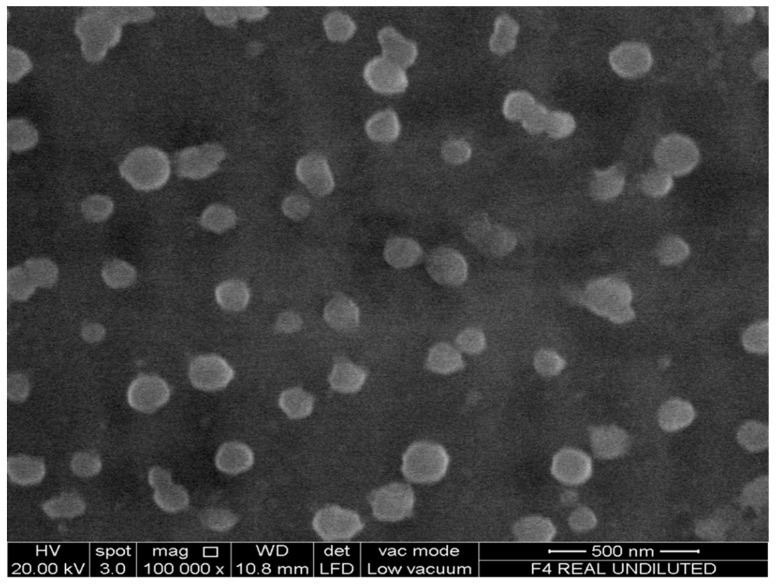
FESEM image of F3PN.

Asantewaa et al., noted that conjugate F3 adsorbed the highest amount of glucose [25], as was the case in a preliminary investigation. Consequently, this conjugate was used to formulate insulin-containing nanoparticles in the present study and labelled as F3PN. These nanoparticles had z-average of 140 ± 12.8 nm, a zeta potential of +19.1 ± 0.69 mV and a pdi of 0.17 ± 0.1. The average diameter of the particles is fairly small and the formulation reasonably homogenous (as indicated by the pdi value). However, the zeta potential is rather low and this can be attributed to the consumption of the –NH_3_^+^ groups in chitosan during conjugation with boronic acid. Nanoparticles with a surface charge of |10| mV are considered approximately neutral, while nanoparticles with zeta potentials of at least |30| mV are considered strongly ionic, thereby ensuring sufficient repulsive forces to keep the particles apart [29]. Notwithstanding, F3PN appear to be well separated from each other as shown in the FESEM image in Figure 1. The particles also appear spherical and in agreement with the z-average determination.

### 3.2. Entrapment of Insulin within Chitosan Nanoparticles 

The drug release profile and pharmaceutical cost-effectiveness of a formulation depend on the drug loading capacity or the encapsulation efficiency (EE%) within the carrier system. This is particularly crucial for nanoparticulate delivery systems which have the smallest size to volume ratio of all dosage forms. There is a range of reported EE% of insulin in nanoparticulate delivery systems. Zhang et al. [30] reported an insulin loading capacity of more than 78% in their polyethylene grafted chitosan nanoparticles, whilst Zhu et al. [31] prepared PEG modified *N*-trimethylaminoethylmethacrylate chitosan nanoparticles which resulted in a range of EE% from 10%–84% depending on the initial weight of the polymer used in the formulation. In the present investigation, an EE% of 81% ± 1.2% was obtained. The PEC method has been used by several researchers to encapsulate insulin in chitosan nanoparticle [7,10,32]. Wu et al., formulated nanoparticles via PEC with an EE% ranging from 49%–59% and contend that this variability was attributed to the amount of insulin used and the molecular weight of the polymer [10]. We may conclude that the EE% obtained in the present investigation is at least comparable to those reported in the literature. The LC% was calculated to be 46±1.8 mg of insulin in 100 mg of nanoparticles.

### 3.3. In Vitro Insulin Release Studies in Various Media

**Figure 2:**
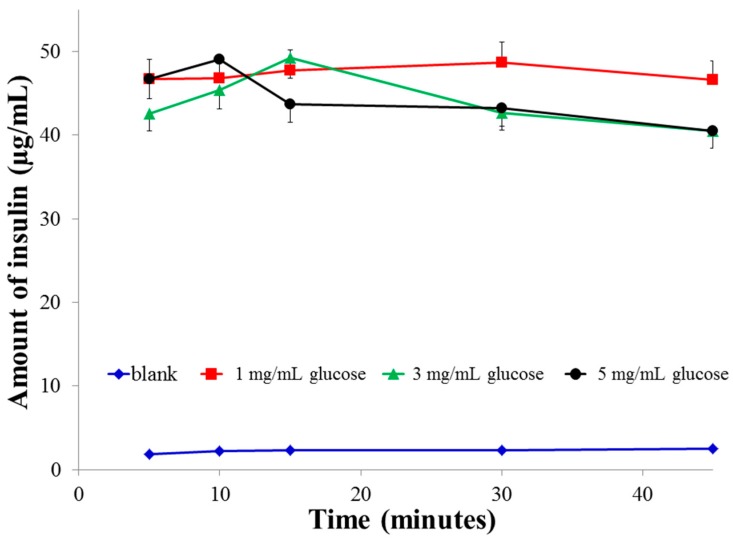
Release profiles of insulin from F3PN in various concentrations of phosphate buffered glucose solution.

Since conjugate F3 adsorbed the highest amount of glucose among all the conjugates and the insulin loading in F3PN was higher than in other reports working on insulin-loaded chitosan nanoparticles, formulation F3PN was used to study the effect of external media (glucose and fructose solutions) on the release of insulin from the nanoparticles. Figure 2 shows the release profile of insulin from F3PN in various concentrations of glucose and blank (no glucose). It can be seen that in 1 mg/mL glucose, the release of insulin remained mostly sustained with a slight peak at 30 min. However, peak insulin release was much earlier (at 15 min) in 3 mg/mL glucose solution which was followed by a dip. In 5 mg/mL glucose, the peak insulin release occurs within 10 min followed by a dip.

The observed phenomena can be attributed to the fact that at higher glucose concentrations, the rate of interaction between diols (glucose) and the boronic acid moieties in the nanoparticles is faster [33] resulting in a faster rate of insulin release which manifested as an early peak. However, no plateau of insulin release was observed in 3 and 5 mg/mL glucose unlike that in 1 mg/mL of glucose solution. Instead, there was a gradual decrease in the concentration of insulin released with time in both these solutions (3 and 5 mg/mL of glucose), eventually reaching a significantly lower concentration (*p* < 0.025) than the final concentration in 1 mg/mL glucose. Therefore, it can be hypothesised that some manifestation causes a slowing of insulin release from the nanoparticles after the initial peaks were attained in both the 3 and 5 mg/mL glucose concentrations. We believe that this manifestation is due to an increase in bi-dendate interactions between the –OH moieties of boronic acid with those of glucose at higher glucose concentrations. This increase in interactions leads to crosslinking of the nanoparticulate matrix by the diols which restricts further insulin release.

**Figure 3:**
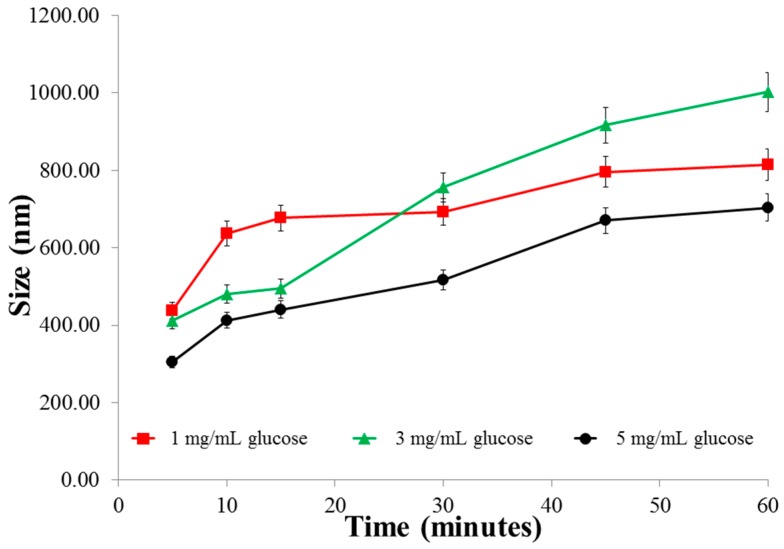
Size changes of the nanoparticles in various concentrations of glucose as a function of time.

The above data correlates with the size changes in various glucose media (Figure 3) where we observe that the size of the particles in 1 mg/mL glucose is largest initially, but then almost levels off after 20 min. In contrast, the size of the particles continues to increase in 3 mg/mL glucose. In 5 mg/mL glucose, the particles remain the smallest after 60 min (significant; *p* < 0.025, when compared to both 1 and 3 mg/mL glucose). This alludes to the possible formation of crosslinking between glucose and boronic acid-conjugated chitosan. The extent and rate of this crosslinking is concentration-dependent and thus explains the fall in insulin release from the nanoparticles in 5 mg/mL glucose (Figure 2). Finally, in the absence of glucose media (blank), there was no trigger for drug release and the nominal amount of insulin released was mainly driven by diffusion forces from the matrix to the medium.

**Figure 4:**
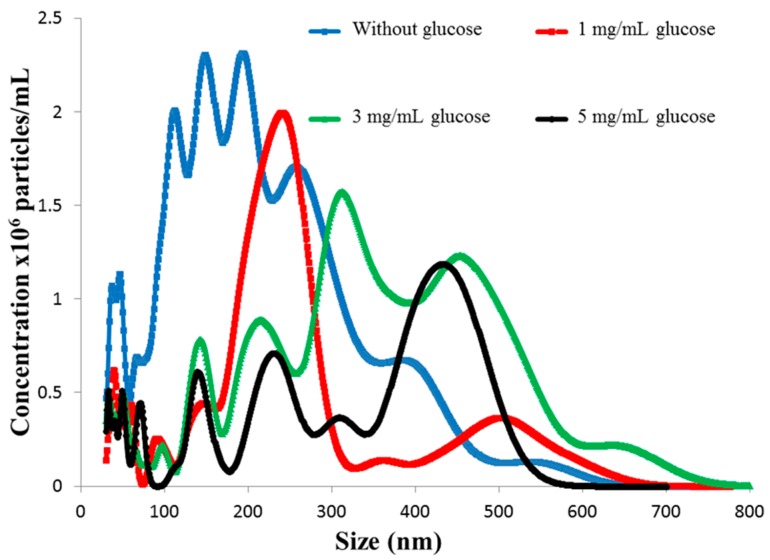
Particle size distribution of F3PN nanoparticles in various concentrations of glucose.

The real-time NTA conducted within 25 min revealed an increase in size of the nanoparticles in the higher glucose concentrations than in the lower glucose concentration. This increase in size of F3PN corroborates with the initial peaks in insulin release at higher glucose concentration observed in 3 and 5 mg/mL glucose. Thus, in Figure 4, it can be noticed that in the absence of glucose, most of the nanoparticles are between 100 and 250 nm, and in 1 mg/mL glucose, the nanoparticles are predominantly between 200 and 400 nm. This implies that there is a glucose concentration-dependent increase in the size of F3PN during the initial stages of exposure. This is a strong corroboration with the data presented in Figure 2. This increase in size is also seen in the higher concentrations of glucose (3 and 5 mg/mL). However, in these two concentrations, about half of the nanoparticles are between 100 and 250 nm as well. This phenomenon further substantiates the theory of crosslinking within the nanoparticulate matrix as proposed earlier, resulting in the particles “shrinking” in size. It is this apparent shrinkage in the size of the nanoparticles that restricts further release of insulin.

**Figure 5:**
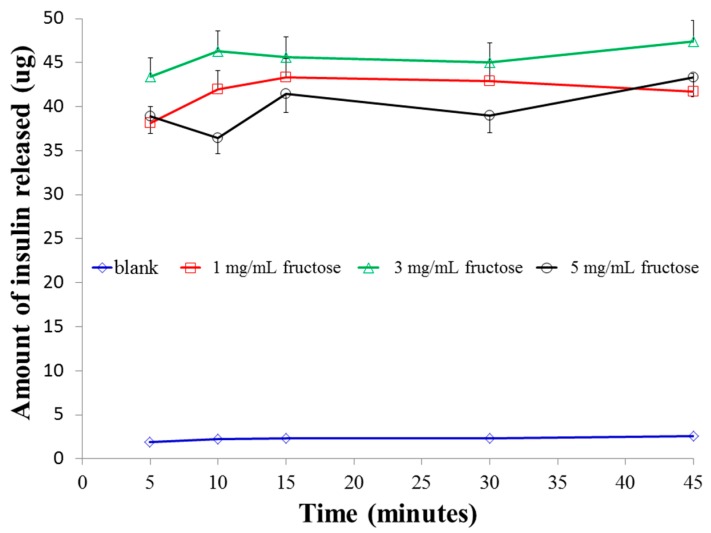
Release profiles of insulin from F3PN in various concentrations of phosphate buffered fructose solutions.

In order to investigate the above observation further, the study was replicated using fructose as an external media. It is well recognized that boronic acids have a significantly higher affinity for fructose than for glucose [33]. This preferential interaction of boronic acid with fructose has been attributed to the lower p*K*_a_ of fructose boronate esters than that of glucose boronate esters. Even though the blood fructose concentration in both diabetic and non-diabetic patients is significantly lower (less than 10 mM) [34] than the blood glucose concentration, we aimed to compare the relative affinities of the boronic acid conjugated chitosan nanoparticles to these two diols at identical concentrations and study how this manifests the crosslinking within the particles. Figure 5 shows the insulin release profiles of F3PN as a function of fructose concentration. In a low fructose concentration (1 mg/mL), there was a sudden release of insulin before a plateau at around 15 min. This pattern of insulin release is identical to that in 1 mg/mL glucose, however the peak insulin release occurs earlier in fructose (15 min against 30 min in glucose), which confirms the superior affinity of boronic acid for fructose. Furthermore, when the concentration of fructose was 3 mg/mL, the final amount of insulin release was significantly higher (*p* < 0.025) than that in 1 mg/mL fructose, which follows from the fact that in 3 mg/mL fructose there is a greater degree of boronic acid-diol complexation than that in 1 mg/mL fructose. Consequently, the polymer matrix swells and releases more insulin (in 3 mg/mL fructose). However, a further increase in fructose concentration (5 mg/mL) resulted in a remarkable decrease (*p* < 0.025 vs. 3 mg/mL glucose; not significant vs. 1 mg/mL glucose, *p* > 0.025) in the total amount of insulin released. This can aptly be attributed to the bidentate interactions between the diols of fructose and that of boronic acids, which effectively crosslinks the nanoparticulate matrix and impedes any further insulin release. The restricted release of insulin in 5 mg/mL of fructose also resulted in an erratic release as shown in the figure. Finally, the peak insulin release in 5 mg/mL fructose is similar to that in glucose of the same concentration.

**Figure 6:**
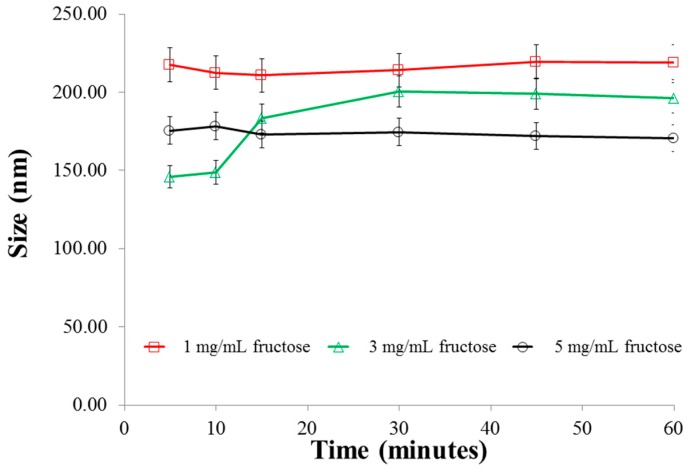
Size changes of the nanoparticles in various concentrations of fructose as a function of time.

Size analysis of the particles in the various fructose media (Figure 6) indicates that in 1 mg/mL of fructose solution, the size of the particles remained almost unchanged, probably due to the fact that in this concentration, an insignificant number of fructose molecules are available for interaction with the boronic acids. Therefore, the amount of insulin released is also largely sustained. In 3 mg/mL, there is an increase in the size of the particles after 10 min and no further increase thereafter. Whist there was no marked change in the size of the particles in 5 mg/mL fructose over the duration of the experiment, the nanoparticles in this concentration of fructose was significantly smaller (*p* < 0.025) than those in 1 and 3 mg/mL fructose. This can be attributed to the strong bidentate interactions between fructose and boronic acid, resulting in the formation of very strong crosslinks within the chitosan polymer chains. This effect resulted in a limited amount of insulin release as presented earlier (Figure 5). Similar to the observation in glucose media (Figure 2), in the absence of fructose media, there was very little release of insulin (less than 3 µg) from F3PNin 45 min.

**Figure 7:**
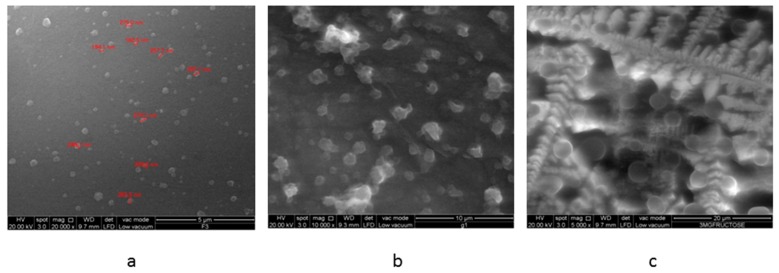
FESEM images of freeze-dried samples of (**a**) freshly prepared F3PN nanoparticulate formulation; (**b**) after exposure to 3 mg/mL glucose; and (**c**) after exposure to 3 mg/mL fructose.

Figure 7a depicts the FESEM image of freshly prepared F3PN nanoparticles loaded with insulin, which appear spherical and uniformly distributed. When the particles were exposed to glucose (Figure 7b), they deformed and aggregated. This appearance is accredited to the non-specific binding nature of glucose to the boronic acid moieties within the chitosan matrix. In this context, it is possible for glucose in its α-d-glucofuranose form, to bind with a pair of OH groups at 1, 2 position [24]. It is also possible for each of the OH groups at positions 3, 5 and 6 of the sugar to bind with one pair of OH groups in boronic acid. We believe that this non-specificity of glucose interaction with boronic acid leads to the formation of distorted crosslinks. Therefore, we observed an initial increase in the nanoparticle size at lower glucose concentrations, which resulted in increased insulin release. However, at higher concentrations of glucose, the release of insulin is slowed by the formation of relatively stronger crosslinks by virtue of the number of the crosslinks mainly. On the other hand, in fructose solution (Figure 7c), we observe that the particles retained their more spherical morphology compared to the nanoparticles in the glucose solution and surrounded by a plethora of crystallized fructose. The binding constant of fructose to boronic acid is significantly higher (4370 M^−1^) than that of glucose (110 M^−1^). Binding between two multivalent (i.e., having more than one binding site) entities involving *n* (*n* > 1) binding events occurs with an affinity higher than the sum of *n* individual monovalent interactions. Most sensing studies using boronic acid have identified fructose as a monovalent ligand with boronic acids binding to its β-d-fructofuranose form [24]. This configuration provides more possibilities for bidentate interactions between the –OH groups of boronic acid with those of fructose. Therefore, the crosslinking within the nanoparticles resulting from these interactions is more regular.

## 4. Conclusions

Release of insulin from the nanoparticles was dependent on the type and concentration of the sugar. There was an initial increase in insulin release which, in higher sugar concentrations, was impeded due to crosslinking of the sugars with boronic acid moieties within the nanoparticles. Due to the higher affinity of boronic acids for fructose, stronger crosslinks were formed within the nanoparticles in fructose media, which resulted in less release of insulin than in glucose media. There is clearly a potential for the delivery system to be developed further to effect a glucose-dependent insulin release feedback capability.

## Figures and Tables

**Table 1:** Variations of 4-formylphenylboronic acid (PBA) used to formulate conjugates [25].

Conjugate	F1	F2	F3	F4	F5
Chitosan (mg)	400	400	400	400	400
PBA (mmol)	0.96	1.92	2.4	4.8	7.2
NaBH_4_ (mg)	240	240	240	240	240
